# The Role of EUPATI CH in Promoting Patient Involvement in Clinical Research: A Multi-Stakeholder Research Project

**DOI:** 10.3389/fmed.2021.795659

**Published:** 2021-12-23

**Authors:** Cristiana Sessa, Caecilia Schmid, Angela Tolotti, Annette Magnin, David Haerry, Loris Bonetti, Ingrid Klingmann

**Affiliations:** ^1^Department of Medical Oncology, Oncology Institute of Southern Switzerland (IOSI), Bellinzona, Switzerland; ^2^Swiss Clinical Trial Organisation (SCTO), Bern, Switzerland; ^3^Department of Nursing, Research and Development Unit, Oncology Institute of Southern Switzerland (IOSI), Bellinzona, Switzerland; ^4^Positive Council Switzerland, Zurich, Switzerland; ^5^European Forum for Good Clinical Practice (EFGCP), Brussels, Belgium

**Keywords:** patient involvement, patient engagement, medicines research and development, drug development, EUPATI, patient representatives, training

## Abstract

**Background:** The European Patients' Academy on Therapeutic Innovation Switzerland (EUPATI CH) was established as an association in 2016 with the mission to improve patient empowerment in Switzerland, raise public awareness of EUPATI's education material, and foster multi-stakeholder partnerships in order to promote public involvement in all aspects of medicines research and development (R&D). In order to achieve its goal of improving patient involvement (PI) in all processes of medicines R&D in Switzerland and to obtain guidance and recommendations for future activities, EUPATI CH initiated a multi-stakeholder survey on PI experiences, hurdles, and best practices. The survey enabled EUPATI CH to obtain and analyze the views of various stakeholders and shape its workplan.

**Methods:** Data collection occurred between January and July 2019 using a survey and semi-structured interviews with individual stakeholders from different groups. The online survey responses were analyzed using quantitative methods and the interviews were analyzed using qualitative methods.

**Results:** The online survey was completed by 55 respondents (10%), and the semi-structured interviews were conducted with 14 stakeholders. Respondents to the online survey were patient representatives (45%), researchers from academia (25%), individuals from the pharmaceutical industry (9%), healthcare professionals (23%), and representatives from government agencies (6%). Some respondents were also members of EUPATI CH. Thirty-eight percent of respondents consider PI in Switzerland to be limited or absent. They identified the main barriers to PI as, first and foremost, a lack of funds and human resources (65%), followed by a lack of information and a lack of education on how to become a patient advocate (21%), a lack of collaboration with other stakeholders (16%), and a lack of adequate resources. Respondents' expectations of EUPATI CH's role in supporting PI were to provide education for active PI and improve networking and collaboration among stakeholders.

**Conclusions:** EUPATI CH's multi-stakeholder research identified some of the difficulties in promoting PI in medicines R&D in Switzerland, in particular the complex collaboration among stakeholders and a lack of funds, human resources, and knowledge. To respond to these difficulties, EUPATI CH has begun preparing a basic training course for patients that is adapted to Switzerland.

## Introduction

Patient involvement (PI) is generally recognized as being valuable when planning health policies that aim to increase their relevance for patients and healthcare. PI has the potential to improve the quality and safety of the healthcare services provided and to increase their value to patients.

There are different ways patients can contribute beyond mere participation, namely as advisors or partners in healthcare research. Besides ethical and political arguments such as democracy and empowerment, there are convincing arguments for the value of PI and patient and public involvement (PPI) in enhancing the relevance, validity, quality, and success of health research.

Involving patients in research can also benefit the medicines development process: bringing in patients' priorities and perspectives can contribute to the development of better treatments for participants and other patients with a particular disease. More active and extensive PI in the medicines R&D process can improve the safety and efficacy of new treatments and can increase the public's awareness of and participation in medical research (1, 2).

The European Medicines Agency (EMA) has been a driver of PPI since 2000. For example, the EMA includes patients in different medicines evaluation committees such as the Committee for Orphan Medicines (COMP) and it promotes the involvement of patients as critical stakeholders in the regulatory process. Over the years, the EMA has developed extensive collaboration with patients and consumer representatives.

The pharmaceutical industry has also recognized the value of integrating patients' contributions into the medicines development, regulatory, and licensing processes; however, it has suffered from a limited availability of patients who are knowledgeable in the relevant methodologies. The Innovative Medicines Initiative (IMI), a public-private partnership between the European Union—represented by the European Commission—and the European Federation for Pharmaceutical Industries (EFPIA), has recognized and supported the need for educating patients on medicines development methodologies and has sought their active involvement as partners in IMI-funded projects. From 2012 to 2017, the IMI ran a pan-European, patient-led project involving 33 public and private organizations with the aim of increasing the education level of patients. This initiative was called the European Patients' Academy on Therapeutic Innovation (EUPATI) project. The main aim of the IMI-EUPATI project was to educate patients using a diploma-type blended learning course and a large toolbox with information material in the seven most spoken languages (3). The EUPATI consortium included patient organizations, academic groups, non-profit organizations, and pharmaceutical companies, and its objectives were to develop and disseminate accessible, well-structured, scientifically reliable educational material for patients that is related to the process of medicines R&D—from the pre-clinical phase to post-marketing. Other topics related to these processes, such as personalized medicine, efficacy and safety assessment, risk-benefit assessment and risk-benefit ratio, health economics, health technology assessment (HTA), and PI were also included. The sustainability plan required by the IMI was successfully implemented in the form of the recently established EUPATI Foundation, which is based in the Netherlands.

EUPATI's concept was based on the experience of HIV and oncology patient communities, in particular the understanding that a better grasp of medicines R&D processes allows patient experts and advocates to work more effectively with the relevant authorities, healthcare professionals, and the pharmaceutical industry—and thus provide valuable input to the medicines development process for the benefit of all patients. Patient experts in patient organizations can become important advocates and advisors in medicines R&D. Gaining relevant expertise can empower patients to provide patient-focused advice and their personal opinions to pharmaceutical industries, academia, authorities, and ethics committees.

In addition to providing training material in the EUPATI Toolbox in now 13 languages, the IMI-EUPATI project has resulted in the establishment of 23 EUPATI National Platforms (ENPs) to date that drive patient education at the national level.

The EUPATI National Platform in Switzerland was established in 2012. In 2016, it was transformed into the association EUPATI CH, with the mission to improve patient empowerment in Switzerland, raise public awareness for EUPATI's education material, and foster multi-stakeholder events to discuss the best pathways for public involvement in all aspects of medicines R&D. In order to achieve its goal of improving PI, to better understand the stakeholders' needs, and to develop guidance and recommendations for the future activities, EUPATI CH started a multi-stakeholder research project in 2019 with semi-structured interviews and a survey. The results of the interviews and survey are reported in this article.

## Methods

### Aims

The overall aim of this multi-stakeholder research project was to obtain guidance and recommendations for EUPATI CH on how to achieve its goal of promoting patient involvement in medicines R&D. Further aims were to identify relevant stakeholders, obtain information about ongoing activities related to PI in Switzerland, identify factors that affect PI, and obtain feedback on the role EUPATI CH can play in improving the impact of PI in the future.

### Study Design

The first steps were to categorize EUPATI CH stakeholders by identifying relevant groups (patients and patient organizations, policymakers and regulators, academia, the pharmaceutical industry, and healthcare professionals) (Appendix 1) and define EUPATI CH's role in PI in medicinal R&D in Switzerland. Stakeholder categories were defined according to the classification of Deverka et al. with some modifications to take into consideration Swiss legislation and the Swiss healthcare system (4).

### Study Population

Stakeholders were categorized based on the information available on their websites or in published material available from their institution or organization. The subsequent step was to establish direct contact with stakeholders to determine their level of knowledge of patient involvement. This was first done by an online survey with target people drawn from lists of existing contacts (EUPATI CH members, participants at EUPATI CH events, and others) according to a convenience sampling (5).

The next step was a personal, semi-structured interview. Interviewees were selected based on a purposing sampling method (6), targeting individuals within each of the five stakeholder groups (patients and patient organizations, health policymakers and regulators, academia, the pharmaceutical industry, and healthcare professionals) who were leaders, people already in contact with EUPATI CH, or expected to have an interest in PI. The interviews were done over the phone or Skype.

### Data Collection

The online survey consisted of 14 questions, seven closed and seven open, on the value of PI, stakeholders' ongoing PI activities, priority areas for each stakeholder group, and their collaboration with the other stakeholders (Appendix 2). In particular, the survey gathered information on the mission of each organization or group, its active involvement of patients, its difficulties and achievements in collaborating with other groups, and major impediments to developing active PI. An additional question provided respondents the opportunity to give their expectations of the role EUPATI CH could or should play in PI.

The interview followed a semi-structured format with six main questions and allowed space for comments and explanations from both the interviewee and the interviewer (Appendix 3). The selected stakeholders received an invitation letter with information on the project, modalities of the interview, confidentiality, and anonymization of the results. The stakeholders who consented to being interviewed received the sample questions at least 2 weeks in advance of the interview. The questions focused on the PI strategy in medicines R&D of the respective stakeholder group/organization, its ongoing activities, difficulties it has faced related to PI, and its future plans for PI. There was an additional optional question on the stakeholder's opinion of EUPATI CH and his or her expectations of EUPATI CH's role in PI in Switzerland.

The interviews were recorded and transcribed verbatim, and the transcriptions were compared with the interviewer's notes. A summary of the transcription was sent to the interviewees for approval to ensure both the accuracy of the overall content of the responses and the internal validity of the results (6). The transcription text itself and the analysis of it were neither checked nor modified by the interviewees.

### Data Analysis

A mixed methods approach was applied.

Answers to the online survey's closed questions were analyzed using a quantitative approach. Answers to closed questions were either on a four-level scale (very important, important, not at all, I don't know), within pre-specified categories, or yes/no and were reported as a percentage of the respondents.

For the interviews a qualitative analysis was performed using a thematic analysis approach (7). This approach involved reading the transcriptions of the interviews, underlining key phrases and phrases that evoked some reflection, and placing them in a list.

This list was used to generate codes (8). This approach allowed EUPATI CH to combine codes matching key phrases from various interviews. Codes could then be combined to derive themes, which could be related to each other. For each category of stakeholder and for each question, the main themes were identified and similar themes from different stakeholder groups were merged into main themes for each question.

The transcriptions of the interviews were analyzed separately by two persons (the interviewer and an expert in qualitative analysis), who then compared the results while referring to the specific parts of the transcription (9). A descriptive comparative analysis of themes among the various stakeholder groups was then performed to extract common and possibly relevant factors that could affect PI.

## Results

### Online Survey

The online survey was sent to 520 stakeholders in January 2019 and was kept open for 4 weeks. The response rate was 10.5% (55/520), and the analyses were performed on a total of 39 evaluable responses. Sixteen responses were not evaluable because answers were either completely missing in 8 cases or the survey was only partially completed in eight additional cases. Table 1 summarizes the responses to the online survey, which are described in detail in the following paragraphs.

**Table 1:** Responses to questions in the online survey.

	**Responses (%)**
Stakeholder category^*^	
Patient organizations	45
Academic research representatives	25
Healthcare professionals	23
Pharmaceutical industry representatives	9
Policymakers and regulators	6
**Current PI limited in medicines R&D**	38
Barriers to PI^*^	
Lack of education	21
Lack of collaboration	16
Language barriers	13
Lack of support	13
Lack of funds and human resources	65
**Priority areas for patients**	
Training and education	60
PI in processes	63
Improving own competence in PI	70
Understanding stakeholders' responsibilities	44
Understanding stakeholders' roles	45
Skills development	40
Guidance, framework, tools	40
Best practices	46

* *Multiple answers possible*.

The largest group of survey respondents were patients (45%), the majority of whom were members of EUPATI CH. They were followed by researchers from academia (25%), healthcare professionals (23%), representatives from the pharmaceutical industry (9%), and policymakers/regulators (6%).

There was almost general agreement on the meaning of PI, which was understood as active collaboration and partnership with all stakeholders while taking into account patients' needs and preferences in the elaboration of projects, the review of protocols, and the composition of advisory boards.

Some 38% of respondents judged their ongoing PI efforts to be limited or absent. A major barrier to meaningful PI in medicines R&D mentioned by respondents was a lack of funds (65%), which was almost always associated with lack of human resources. Other barriers mentioned were a lack of education and/or information on this particular topic and on how to become a patient advocate (21%), a lack of collaboration with stakeholders, in particular with academic institutions (16%), difficulty in finding suitable patients due to language barriers (13%), a lack of networking together with a lack of lobbying and support from existing structures (13%). The following impediments were also identified: PI in R&D is not the main focus of an organization, a lack of awareness of PI by key actors (hospitals, medical faculties), difficulties in reaching the experts, fear, and skepticism.

When asked to rate priorities, patients rated the following areas the highest: being involved in processes (63%), receiving training and education (60%), and understanding different stakeholders' responsibilities (44%). In addition, 70% of patient respondents would like to improve their capabilities in or knowledge of PI, in particular how to ensure reliability, stability, and interaction with patient groups, how to interact with stakeholders, and how to spread awareness of PI as a person and as a society.

### Face-to-Face Interviews

Table 2 presents the response rates to the invitation for a face–to-face interview for each group of stakeholders. Fourteen interviews, with an average duration of 45 min (range: 30–55 min.), were conducted and analyzed. Quotes from interviewees that are used in this article are presented in italics and within quotation marks so they can be easily identified. A superscript number indicates which quote from an interviewee a specific statement corresponds to (Appendix 4).

**Table 2:** Face-to-face interviews: Qualitative analysis per stakeholder group.

**Stakeholder group**	**Contacted**	**Accepted interviews**	**Qualitative analysis**
Patient organizations	8	4^*^	4
Academia	6	5^**^	5
Policymakers and regulators	3	2^***^	2
Pharmaceutical industry	4	3^**^	3
	20	14	14

* *2 never replied, 1 was too busy, 1 interview was cancelled and only written text was provided by the organization*.

** *1 never replied*.

*** *1 not allowed*.

### Stakeholders' Patient Involvement Strategies, Ongoing Activities, and Successes

In terms of strategies—either implemented or planned—to increase PI in medicines R&D, by patient organizations reported increasing collaboration with other stakeholders as a strategy (Table 3A). For small patient organizations, it was very important to have a “united voice” with other patient organizations when collaborating with other stakeholders. Collaborating with the pharmaceutical industry was also an important strategy for patient organizations because it facilitated reimbursement, access to active compounds, and research on new drugs (1). Collaborating with some regulators was experienced as being difficult; however, the value of being in contact with both regulators and health authorities was generally recognized.

**Table 3A:** Qualitative analysis: Main themes for respondents from patient organizations and in academia.

	**Strategies**	**Ongoing activities**	**Successes**	**Difficulties**	**Future activities**
**Stakeholder group (n:4)**				
Patient organizations (2 small, 2 large)	•↑ Collaboration with SKs •Educated pts: 1) Make informed decision 2) ↑ Awareness pts rights 3) Speak effectively •Direct PI (boards, events, communication, data collection)	•Data collection, scientific boards •Online strategy •Definition of objectives of future research •Organization of events	•↑ SK awareness •Good collaboration with pharma and some SKs (clinicians, pt orgs)	•With members •Other SKs (mainly authorities and other pt orgs) •Lack of funding •↑ Complexity of disease and treatment	•↑ Collaboration with authorities and other pt orgs •Enable pts to become active/train to be experts •↑ External communication activities
**Stakeholder group (n:2)**					
Academia	•Direct PI •↑ PI by: 1) ↑Awareness 2) Making it a high-priority program	•PI as evaluation criteria for academic studies •Building up network of the society •Direct PI in: 1) Registry for major problems 2) Focus groups 3) Boards	Too early	•Collaboration with pts/parents: 1) How to contact them 2) How to find the proper representative 3) Lack of time 4) Little scientific knowledge: •Highly specialized studies •Small, disease-specific orgs •Trial failures due to low feasibility, pt accrual	•↑ PI by: 1) Looking for parents interested in research 2) Direct involvement in projects 3) ↑ Funding •Develop research in the community

*SK, stakeholder; pt, patient; PI, patient involvement; EC, ethics committee; WG, working group; GC, general consent; DB, database; orgs, organizations*.

For some large patient organizations, collaborating with other patient organizations was not part of their main strategy because collaboration could result in heterogeneous objectives and create confusion. Some of the larger organizations do not collaborate with the pharmaceutical industry in order to maintain financial independence (2), which was also reported as one of the difficulties encountered by stakeholders in the pharmaceutical industry (Table 3B). The pharmaceutical industry aims to increase its collaboration with large patient organizations (either European or national) and their local affiliates in order to establish contact with patients or patient organizations and establish a long-term collaboration as equal partners.

**Table 3B:** Qualitative analysis: Main themes for policymakers and regulators and respondents in the pharmaceutical industry.

	**Strategies**	**Ongoing activities**	**Successes**	**Difficulties**	**Future activities**
**Stakeholder group (n:2)**					
Policymakers and regulators	•Involve pts in ECs •↑ Information for pts/laypeople	•Educational programs for new EC members •Collaboration with health leagues	•Good contact with pt orgs •↑ pts' attention to e-health literacy	•Poor acceptance of GC •Heterogeneity of pt orgs •Reluctance of some SKs to involve pts	•List of pts to be contacted •Research on best GC •↑ PI in e-health information
**Stakeholder group (n:2)**					
Pharmaceutical industry (n:3)	•Global strategy: pt-dedicated teams and local representatives •Increase: 1. Knowledge of pt advocacy 2. Pt empowerment through education 3. Equality in partnership with pts	•Involve local pt representatives •Bring pts' view into whole life cycle •Long-term teaching in collaboration with EU umbrella orgs •Use pt advocates to empower pts	•↑ PI •Ongoing community studies •Involvement in projects of international pt orgs	•Change is too slow in industry •Collaboration with some pt organizations •Pharma industry's bad reputation	•Cover all activities in development •Increase internal PI awareness •Organize events to bring together pt orgs.

*SK, stakeholder; pt, patient; PI, patient involvement; EC, ethics committee; GC, general consent; EU, European Union; orgs, organizations*.

Patient education is a very important activity for patient organizations. Education “*like EUPATI”* mainly covers the whole life cycle of a product, but some members have also requested training on how to improve their public communication skills (3). Some patients would like to be more involved in discussions with authorities such as Switzerland's Federal Office of Public Health (FOPH), but they cannot because of their perceived lack of competence. Training provided by EUPATI could represent a great opportunity for patients to achieve a more active, direct role in an organization (4). Patient education is also one of the pharmaceutical industry's strategies, in particular in collaboration with EU umbrella patient organizations for long-term teaching; related activities are already underway (Table 3B). It is part of a global strategy to have teams dedicated to increasing patient empowerment and integrating patients' views in all phases of the medicines life cycle.

From both academia's and patient organizations' perspectives, direct PI was reported as an important strategy in a variety of activities (e.g., the organization of events like patients' day, scientific boards, data collection, and focus groups). Specific activities varied according to the needs and mission of an organization or institution.

In the academic setting, a reported strategy for promoting PI entailed raising physicians' awareness of PI, setting PI as a high priority topic for the next 5 years, and raising patients' awareness of PI by reporting study results or research activities in social media. Patients also play a major role in the collection of personal data in prospective registries and in the evaluation of quality of life (QoL) questionnaires for the purpose of developing tools that are able to evaluate the real burden of symptoms relevant to patients (5).

Patients participate in focus groups, sometimes led by the pharmaceutical industry, and are on a variety of boards, such as scientific boards that evaluate clinical study proposals. One respondent noted, “*It's important to have people living with a disease included in research decisions on what research is funded.”*

### Difficulties Stakeholders Faced With Patient Involvement

All patient organizations interviewed reported difficulties interacting with the authorities, in particular with Switzerland's Federal Office of Public Health (FOPH). One patient expressed his concern about the lack of PI and the lack of control at the regulatory level on the upcoming availability of effective personalized treatments that patients do not have guaranteed access to (6). A lack of funding and human resources applies to almost all stakeholders, in particular small patient organizations. This impedes hiring additional personnel and implementing new programs and activities (7).

Difficulties working with patients occur in a variety of situations according to various stakeholder groups. For patient organizations, one difficulty is how to actively involve members because they need to be instructed on how to perform tasks and require support by a dedicated person. One patient organization reported difficulty finding patient experts willing to assess many research projects (8) and difficulty representing a more general patient perspective—a situation also observed by regulators interested in the contributions of patients who are members of ethics committees.

One academic representative mentioned that it is difficult to find parents for pediatric studies who are interested in research beyond their own children. In addition, some parents have very little knowledge of rules, regulations, and limitations, which decreases the value of their participation on boards. Despite these difficulties, stakeholders want to have patients on boards so they can share their experience, perspectives, and needs.

Pharmaceutical companies' main difficulties were improving the internal appreciation of the value of PI and overcoming an external negative reputation due to previous questionable behavior, a factor that affects collaboration with some patient organizations. For regulators, the main difficulties were the poor acceptance of general consent (GC) and the heterogeneity of patient organizations, which makes it difficult to find educated patients willing to serve as patient representatives in an ethics committee.

### Stakeholders' Future Patient Involvement Activities

Patient organizations plan to direct some of their future activities at further improving ongoing initiatives, in particular those with authorities, as well as increasing patient education (9) “*because being active as a patient has a direct benefit for ourselves”* and developing external communication and networking (Table 3A).

In academia, future activities will be aimed at improving PI in research as well as improving collaboration with patient organizations. Future activities will also focus on developing a stronger link between university hospitals and the community in order to explore the possibility of addressing questions that are more important to the community than to the university hospitals (10).

For the pharmaceutical industry, future activities will be directed at involving patients in all activities of medicines development and organizing events to bring patient organizations together.

## Stakeholders' Comments Directed At EUPATI CH

### Desired Activities

More EUPATI patient training was one the activities most desired by patient organizations, academia, and the pharmaceutical industry, as mentioned in both the online survey and the direct interviews (Table 4). For academia, it would also be helpful to have EUPATI training to increase interaction with EUPATI CH and the use of its toolbox material (11).

**Table 4:** Qualitative analysis of main themes related to EUPATI CH: Desired activities, criticisms, and its expected role.

**Desired activities**
EUPATI CH should provide: •Training for patients (pt orgs, A) and the pharmaceutical industry (P) •Networking with other patient organizations (pt orgs, R, A) •Information to patients (pt orgs), the community (R), and academia (A) •A shared opinion on questions related to patients in clinical research (R)
**Criticisms**
•Lack of clarity regarding mission (P) •Unfulfilled tasks (P) •EUPATI CH's financial support from the pharmaceutical industry (R, A)
**Expected role of EUPATI CH**
•Connect with patient advocates (P) •Run multi-stakeholder initiatives to support patients/the community (P, pt orgs)

*pt orgs, patient organizations; P, pharmaceutical industry; A, academia; R, policymakers and regulators*.

Another desired activity, also mentioned in both the online survey and the direct interviews, was support for PI promotion and interaction among patient groups, with EUPATI “*being a good neutral platform where the patient groups get together*.” For regulators, EUPATI CH can find shared opinions on questions of national relevance, for example general consent (GC) or the patient's role in ethics committees, and can bring patient information from the European level to the specific national needs of an organization (12).

### Criticisms and Expected Tasks

One criticism of EUPATI CH, raised by a pharmaceutical company representative, was the lack of clarity on EUPATI CH's mission and objectives in relation to those of EUPATI at the European level that EUPATI CH strives to adapt and implement (Table 4). EUPATI's mission is to offer patient training and thus empower and connect patient advocates as well as scale up know-how in organizations and people. The pharmaceutical industry representative criticized EUPATI CH for not having done this yet [13].

Another criticism made by some academic respondents was the lack of transparency regarding EUPATI CH's funding. “*It's important for your credibility that you can demonstrate where the funding is coming from.”* The IMI-EUPATI project was set up as a public-private partnership, whereas EUPATI CH is a private association. Nevertheless, regulators raised the concern that EUPATI CH could be partly financed by industry and thus have a potential conflict of interest [14].

## Discussion

EUPATI CH undertook this multi-stakeholder project in order to obtain recommendations for improving its activities related to the promotion of PI in medicines R&D in Switzerland.

For the performance of the study we applied a mixed-method design to get a more complete understanding of the phenomenon and hear the voices of Eupati CH stakeholders (12).

In the small, selected population surveyed, PI in medicines R&D was judged to be limited or absent by 38% of respondents. For patient organizations, the qualitative analysis clarified some aspects of the main impediments (lack of funds, lack of human resources and knowledge, lack of interactions with other stakeholders) which had been already reported in the quantitative analysis.

The respondents identified the main impediments to PI in medicines R&D in Switzerland as lack of funds and human resources (65%), lack of knowledge and capabilities (21%),lack of collaboration (16%). The qualitative analysis confirmed those results and further defined the characteristics of the impediments (Tables 3A,B).

There is a general agreement on the relevance of information on the value of a direct PI in clinical research and of an increased collaboration among different stakeholders. Additional points are the lack of funds and human resources. For patients organizations, there is a specific need for training to become an active expert and to increase the collaboration with other patient organizations and authorities.

One important success of EUPATI was, at least for small organizations, the possibility to collaborate with academia through direct involvement in the preparation and organization of clinical studies. This is a success because it confers a primary role to patients and leads to the improvement of clinical research and patient care. As one patient stated, “*You cannot have a successful project if you are not also taking into account patients' needs.”*

Stakeholders' opinions on the opportunities and benefits of collaborating with the pharmaceutical industry were divergent and seemed to be dependent, at least partly, on the size, financial resources, and availability of an effective treatment as well as the terms of the collaboration.

The difficulties related to PI in medicines R&D that were identified in EUPATI CH's survey have also been documented in other countries. For example, a lack of funding and available time to support panel members and patient organizations, tension between various stakeholder groups when developing and conducting clinical research, and concern related to the level of patients' and the public's understanding of certain types of research were the main difficulties identified when a PPI model was implemented in cancer and palliative care in the United Kingdom (10).

The limitations of the present evaluation are the small sample size and the favorable selection of the population studied, which potentially reduces the transferability of the results. Potential reasons for the poor response to the survey were declared lack of interest, survey fatigue, lack of knowledge of EUPATI, difficulties in identifying the person responsible of patient involvement in Switzerland within large organizations The innovative aspect is the application of a qualitative analysis of stakeholders' opinions and comments, thus bringing in the voice of patients as well as public opinion in two complementary surveys: one online and one as direct interviews. This dual approach helped to clarify some of the features of the data collected in the online survey.

### Recommendations for Future EUPATI CH Activities

EUPATI's competence in education and training was appreciated by all stakeholder groups. Besides education on medicines R&D, stakeholders requested that EUPATI CH teach communication skills in order to improve direct interactions between patients and regulatory bodies.

Generally, stakeholders support EUPATI CH's collaboration with pharmaceutical companies in education and training but think that it should first be discussed and clarified in terms of content, modalities, audience, scientific freedom, and the role of EUPATI CH.

Another role EUPATI CH could assume is that of a neutral national platform that fosters multi-stakeholder events, channels patient-relevant information from the European level to the national level, and facilitates networking.

The need for multi-stakeholder collaboration to improve PI in healthcare is also the conclusion of a survey conducted by EUPATI BE, a platform for patient education established in 2017 as EUPATI's National Platform in Belgium (11). Its survey was conducted on different stakeholder groups (academic stakeholders, patient organizations, patients, industry, and policymakers) than those in our study. The major barriers to PI identified in EUPATI BE's survey were a lack of information and education, the lack of a favorable regulatory and ethics environment, a lack of PI awareness, low levels of communication and trust, and the lack of a systematic and structured approach. In all these areas, EUPATI and its national platforms could play a strategic and proactive role in the future.

Respondents' criticisms of EUPATI CH are useful for highlighting weaknesses in EUPATI CH's activities so far and for identifying activities that should be implemented in the near future. The lack of clarity regarding EUPATI CH's mission may be partly due to the limited extent and lack of clarity of information that EUPATI CH has distributed, but it could also be related to a lack of common focus in EUPATI CH's activities.

To address these criticisms, EUPATI CH needs to take a more systematic and structured approach to PI in order for its PI efforts to be efficient and effective. In addition, adequate funding, transparency, codes of conduct for all involved stakeholders, and overarching policies are needed. Another step EUPATI CH should take is to prepare clear, straightforward information on its mission, structure, and financial support as well as its relationship with pharmaceutical companies. This information can then be distributed through various platforms, communication channels, and social media. Other recommendations EUPATI CH can act on in order to fulfill its mission to improve patient empowerment are to provide more PI education and host multi-stakeholder events. With this in mind, EUPATI CH is currently preparing a training course for patients and patient representatives that aims to teach them the fundamentals of clinical research and how these fundamentals apply within the context of legal and ethical requirements in Switzerland.

From a more general perspective, an increase awareness of the community on the value and benefit of a direct involvement of patients in healthcare research should be pursued and supported by the different stakeholders to become an important component of clinical practice.

## Data Availability Statement

The original contributions presented in the study are included in the article/Supplementary Material, further inquiries can be directed to the corresponding author.

## Ethics Statement

Ethical review and approval was not required for the study on human participants in accordance with the local legislation and institutional requirements. Written informed consent for participation was not required for this study in accordance with the national legislation and the institutional requirements.

## Author Contributions

CSe was responsible for the academic design and drafted the article with CSc, AM, and DH. IK substantially revised the article and DH provided critical insight and text revision. CSe was responsible for the study design, data analysis, and data interpretation. CSc was responsible for the design and preparation of the online survey. AT and LB performed the qualitative analysis of data and interpretation. All authors read and approved the final manuscript.

## Funding

EUPATI CH funded this study through an unrestricted grant from Amgen.

## Conflict of Interest

The authors declare that the research was conducted in the absence of any commercial or financial relationships that could be construed as a potential conflict of interest.

## Publisher's Note

All claims expressed in this article are solely those of the authors and do not necessarily represent those of their affiliated organizations, or those of the publisher, the editors and the reviewers. Any product that may be evaluated in this article, or claim that may be made by its manufacturer, is not guaranteed or endorsed by the publisher.

## References

[1] Buck D Gamble C Dudley L Preston J Hanley B Williamson PR. From plans to actions in patient and public involvement: qualitative study of documented plans and the accounts of researchers and patients sampled from a cohort of clinical trials. BMJ Open. (2014) 4:e006400. 10.1136/bmjopen-2014-006400 25475243 PMC4256646

[2] Stewart R Liabo K. Involvement in research without compromising research quality. J Health Serv Res Policy. (2012) 17:248–51. 10.1258/jhsrp.2012.011086 22814591

[3] EUPATI. Available online at: https://www.eupati.eu/closing-report-eupati-2012-2017/ (accessed December 31, 2017).

[4] Deverka PA Lavallee DC Desai PJ Esmail LC Ramsey SD Veenstra DL. Stakeholder participation in comparative effectiveness research: defining a framework for effective engagement. J Comp Eff Res. (2012) 1:181–94. 10.2217/cer.12.7 22707880 PMC3371639

[5] Morse JM. Critical analysis of strategies for determining rigor in qualitative inquiry. Qual Health Res. (2015) 25:1212–22. 10.1177/1049732315588501 26184336

[6] O'Brien BC Harris IB Beckman TJ Reed DA Cook DA. Standards for reporting qualitative research: a synthesis of recommendations. Acad Med. (2014) 89:1245–51. 10.1097/ACM.0000000000000388 24979285

[7] Braun V Clarke V. Using thematic analysis in psychology. Qual Res in Psychol. (2006) 3:77–101. 10.1191/1478088706qp063oa32100154

[8] McIntosh MJ Morse JM. Situating and constructing diversity in semi structured interviews. Glob Qualit Nurses Res. (2015) 2:1–12. 10.1177/2333393615597674 28462313 PMC5342650

[9] Holloway I Wheeler S. Qualitative Research in Nursing and Healthcare. Hoboken, NJ: Blackwell Publishing Ltd. (2010).

[10] Collins K Boote J Ardron D Gath J Green T Ahmedzai SH. Making patient and public involvement in cancer and palliative care research a reality: academic support is vital for success. BMJ Supp Pall Care. (2015) 5:203–6. 10.1136/bmjspcare-2014-000750 25252940

[11] Grine L Janssens R van Overbeeke E Derijcke D Silva M Delys B. Improving patient involvement in the lifecycle of medicines: insights from the EUPATI BE survey. Front Med. (2020) 7:36. 10.3389/fmed.2020.00036 32118020 PMC7031274

[12] Fetters MD Curry LA Creswell JW. Achieving integration in mixed methods designs-principles and practices. Health Serv Res. (2013) 48(6 Pt 2):2134–56. 10.1111/1475-6773.12117 24279835 PMC4097839

