# Supplementary material for: The Role of EUPATI CH in Promoting Patient Involvement in Clinical Research: A Multi-Stakeholder Research Project
Source: Front Med (Lausanne). 2021 Dec 23;8:795659. doi: 10.3389/fmed.2021.795659 (PMC8733300; doi:10.3389/fmed.2021.795659)
Supplement: Supplementary file 1 [file Data_Sheet_1.pdf]

Appendix 1. Stakeholder categories

Stakeholder category	Description
Patients and patient organizations	Persons or organizations that represent patients' perspectives generally or within a specific disease, for example patients, patients' relatives, patient advocates, or patient advocacy organizations
Policymakers and regulators	Individuals in organizations or institutions that create, oversee, and monitor policies or regulations of healthcare-related issues, for example government institutions or organizations and clinical guidelines developers.
Academia	Non-profit entities or research institutions that develop and provide scientific knowledge and clinical products or monetary and structural support for research efforts, for example non-profit foundations.
Pharmaceutical industry	Companies and organizations in industry that discover, develop, produce, and market medications or medical devices for patients in order to cure, vaccinate, or improve well-being.
Healthcare professionals	Individuals who provide healthcare services, for example physicians, nurses, pharmacists, nurse practitioners, physician assistants, and mental health providers.

Appendix 2. Online survey

1. Which category of stakeholder best defines your organisation or group? (multiple answers allowed)
    a. Patient (Advocacy) Organisations / individual patient advocate (representative)
    b. Academia (Researchers)
    c. Policy-makers / regulators
    d. Pharmaceutical Industry
    e. Healthcare Professionals
    f. Payers and Purchasers
    g. EUPATI CH member
    h. Other (please specify) (add freetext box)

2. What is the mission of your organisation? Please also provide if applicable the link to your organisation`s website.
    a. (Freetext box)

3. What does "patient involvement in medicines R&D" mean to you?
    a. (Freetext box)

4. Is patient involvement in medicines R&D important for your group / yourself?

	Very important	Important	Not at all	I don't know
How important is it now	○	○	○	○
How important should it be, in future	○	○	○	○

Why or why not is it important to you?

5. What is your stakeholder group's role in patient involvement in medicines R&D? Is there any form of patient involvement in your group/organisation?
    a. (Freetext box)

6. Which are your perceived (and real) barriers to a meaningful patient involvement in medicines R&D?
    a. (Freetext box)

7. With which stakeholder groups are you currently working/collaborating, or have you worked in the past?
    a. None
    b. Patient (Advocacy) Organisations / individual patient advocate (representative)
    c. Academia (Researchers)
    d. Policy-makers / regulators
    e. Pharmaceutical Industry
    f. Healthcare Professionals

g. Payers and Purchasers
h. EUPATI CH member
i. Other (please specify) (freetextbox)

8. Is your collaboration with other stakeholder groups with regard to patient involvement effective? What works well? / What does not work so well?
   a. (freetextbox)

9. Which are your priority areas, in relation to patient involvement in medicines R&D? Please rate

	Very much	Likely	Not at all	I don't know
Training and education	○	○	○	○
Skills development	○	○	○	○
Understanding responsibilities	○	○	○	○
Clarifying / understanding the role in R&D	○	○	○	○
Guidance, framework, tools	○	○	○	○
Best practices, proof of benefit / value	○	○	○	○
Getting patient involved in the processes (decision making, research projects, boards, …)	○	○	○	○

Other (specify)

[ ]

10. Are you performing educational/awareness activities related to patient involvement in medicines R&D?
    a. Yes
    b. No
    c. If not, why? (freetext box)

11. Are there capabilities or knowledge in this field you would like to build on?
    a. Yes
    b. No
    c. Please describe (freetext box)

12. Is the lack of adequate resources a major impediment to your group's activities in patient involvement in medicines R&D? Please specify
    a. Lack of funds
    b. Lack of human resources
    c. Lack of knowledge, capabilities
    d. Other (please specify)

13. What are your expectations towards EUPATI Switzerland and what role should it play in the patient involvement within the country?
    a. (freetext)

14. How can you contribute to EUPATI CH and get involved with us?
    a. (freetext)

15. Your contribution is very much valued, would you like to receive the summary of this survey? Then please provide your contact details
    a. Name
    b. Organisation
    c. Email Address

Appendix 3. Interview questions

1. Do you have strategies and objectives in patient involvement? Is there a person in charge of it?

2. Which are the activities and the processes for involving patients and carers already ongoing in your group/organization?
   If there aren't, why not?
   If yes, are they part of the strategy?

3. Please comment on the results already achieved, are you satisfied?

4. Which were the main difficulties so far encountered? Were they expected? Why?

5. What are you going to do next?

6. Have you ever looked at products/services and how patient involvement could improve your output?

7. Do you have any other comment on patient involvement?

Appendix 4. Original quotes from interviewees

No.	Quotes	Corresponding wording in this article
1	"We are doing a lot of work with pharmaceutical industries."<br><br>"They [pharmaceutical companies] are present to a certain degree; we are partners in a process."<br><br>"… because they are the ones with medications for research and everything."	Collaborating with the pharmaceutical industry was also an important strategy for patient organizations because it facilitated reimbursement, access to active compounds, and research on new drugs.
2	"We are not working with a certain type of industry"<br><br>"It [our collaboration with the pharmaceutical industry] is really disconnected—it's no longer an exchange of money."	Some of the larger organizations do not collaborate with the pharmaceutical industry in order to maintain financial independence.
3	"The second priority is to educate patients to make informed decisions."<br><br>"[education] like EUPATI"<br><br>"We do quite a lot of internal work to educate our members."<br><br>"We have a lot of members."	Patient education is a very important activity for patient organizations. Education "like EUPATI" mainly covers the whole life cycle of a product, but some members also requested training on how to improve their public communication skills.
4	"… and this [having a more active role in an organization] would also promote our members."	Some patients would like to be involved more in discussions with authorities such as Switzerland's Federal Office of Public Health (FOPH), but they cannot because of their perceived lack of competence. Training provided by EUPATI could represent a great opportunity for patients to achieve a more active, direct role in an organization.
5	"We try to bring our voice in. We want to make sure that fatigue and also pain are captured when direct patient involvement is also promoted by the pharmaceutical industry."	Patients also play a major role in the collection of personal data in prospective registries and in the evaluation of quality of life (QoL) questionnaires for the purpose of developing tools that are able to evaluate the real burden of the symptoms relevant to patients.

6	"The problem which might come to us can be very big. Personalized therapies are there, but nobody can pay for them. They just let [the] pharmaceutical industry do and look what is happening. It's so important for the patient—they should have steering power … to ensure the patient can be really involved in this development in the next 10–15 years."	One patient expressed his concern about the lack of PI and the lack of control at the regulatory level on the upcoming availability of effective personalized treatments that patients do not have guaranteed access to.
7	"If we had more money, we could hire another person who can do other works … and we would have many important things to do."	This impedes hiring additional personnel and implementing new programs and activities.
8	"Because if you want members to actively contribute, you have to indicate the area of work they should focus on and you need dedicated people to follow them"<br><br>"It's also quite hard to find patient experts, and it's a lot of work to assess many research projects."	For patient organizations, one difficulty is how to actively involve members because they need to be instructed on how to perform tasks and require support by a dedicated person. One patient organization reported difficulty finding patient experts willing to assess many research projects …
9	"… tackle many of the difficulties just mentioned, especially those with authorities. It's very important to me that they hopefully in some years understand something about you."<br><br>"I dream of a world of very informed patients."<br><br>"Enabling people to become active would be something very great."	… improving ongoing activities, in particular those encountered with authorities, as well as increasing patient education …
10	"[Because there] we can address questions maybe more important in the community than in the university hospitals."	… developing a stronger link between university hospitals and the community in order to explore the possibility of addressing questions that are more important to the community than to the university hospitals.
11	"… have this training from EUPATI"<br><br>"I'll have to invite them to present also things that we can use from the website."<br><br>"We have to discuss how to interact better and use each other as [a] network."	For academia, it would also be helpful to have EUPATI training to increase interaction with EUPATI CH and the use of its toolbox material.
12	"EUPATI can bring an information flow from the European level to the Swiss-specific needs of the specific organization."	… can bring patient information from the European level to the

		specific national needs of an organization.
13	"Doing something that's not EUPATI above the market level: EUPATI is not a patient organization but an empowerment organization."<br><br>"EUPATI stands for training, empowerment, upskilling organizations and people—that it is not yet done."	EUPATI's mission is to offer patient training and thus empower and connect patient advocates as well as scale up know-how in organizations and people … criticized EUPATI CH for not having done this yet.
14	"I heard from some people … that EUPATI is financed partly by the industry. They think that there is also, like, a conflict of interest."<br><br>"Small patient organization[s] don't have a financial basis, but they don't want, like EUPATI, [to] work together with [the] pharmaceutical industry."	… regulators raised the concern that EUPATI CH could be partly financed by industry and thus have a potential conflict of interest.
